# The Art of Body Movement: Health Impact on Older Adults With Disability and Dementia

**DOI:** 10.1093/geroni/igaa057.1875

**Published:** 2020-12-16

**Authors:** Kuei-Min Chen

**Affiliations:** Kaohsiung Medical University, Kaohsiung City, Taiwan (Republic of China)

## Abstract

Body-movement is an art form of self-expression and health promotion. This presentation will provide an overview of studies that employ the use of “movement activities” or “exercise” as non-pharmacological modalities to improve activities of daily living and functional fitness (e.g., cardiopulmonary function, body flexibility, range of joint motion, and muscle strength and endurance) of older adults with disability. These studies have also been shown to decrease depression state and behavior symptoms of older adults with dementia in long-term care facilities. Moreover, the Wheelchair-bound Senior Elastic Band (WSEB) exercise program will be described that are of relevance to the older adults with disability and dementia.

